# Bruton’s Tyrosine Kinase (BTK) Inhibitor RN486 Overcomes ABCB1-Mediated Multidrug Resistance in Cancer Cells

**DOI:** 10.3389/fcell.2020.00865

**Published:** 2020-08-27

**Authors:** Xing-Duo Dong, Meng Zhang, Xiubin Ma, Jing-Quan Wang, Zi-Ning Lei, Qiu-Xu Teng, Yi-Dong Li, Lusheng Lin, Weiguo Feng, Zhe-Sheng Chen

**Affiliations:** ^1^Department of Pharmaceutical Sciences, College of Pharmacy and Health Sciences, St. John’s University, Queens, NY, United States; ^2^First Clinical College, Shandong University of Traditional Chinese Medicine, Jinan, China; ^3^Cell Research Center, Shenzhen Bolun Institute of Biotechnology, Shenzhen, China; ^4^College of Bioscience and Technology, Weifang Medical University, Weifang, China

**Keywords:** RN486, Bruton’s tyrosine kinase inhibitor, multidrug resistance, ATP-binding cassette transporter, ABCB1

## Abstract

Overexpression of ATP-binding cassette subfamily B member 1 (ABCB1) remains one of the most vital factors leading to multidrug resistance (MDR). It is important to enhance the effect and bioavailability of chemotherapeutic drugs that are substrates of ABCB1 transporter in ABCB1-overexpression cancer cells and reverse ABCB1-mediated MDR. Previous, we uncovered that the Bruton’s tyrosine kinase (BTK) inhibitor ibrutinib is a potent reversal agent to overcomes paclitaxel resistance in ABCB1-overexpressing cells and tumors. In this study, we explored whether RN486, another BTK inhibitor, was competent to surmount ABCB1-mediated MDR and promote relevant cancer chemotherapy. We found that RN486 significantly increased the efficacy of paclitaxel and doxorubicin in both drug-selected carcinoma cells and transfected cells overexpressing ABCB1. Mechanistic studies indicated that RN486 dramatically attenuated the drug efflux activity of ABCB1 transporter without altering its expression level or subcellular localization. The ATPase activity of ABCB1 transporter was not affected by low concentrations but stimulated by high concentrations of RN486. Moreover, an interaction between RN486 with ABCB1 substrate-binding and inhibitor binding sites was verified by *in silico* docking simulation. The results from our study suggest that RN486 could be a reversal agent and could be used in the novel combination therapy with other antineoplastic drugs to conquer MDR-mediated by ABCB1 transporter in clinics.

## Introduction

Uncovering the best treatment for cancer is still challenging since cancer is one of the most dreadful diseases for the last several decades (Roy and Saikia, 2016). Chemotherapy is one of the primary and vital treatments to fight with a variety of cancers (Zhang et al., 2015). However, the presence of multidrug resistance (MDR) decreases the efficacy of anticancer drugs (Wu et al., 2011). It has been found that the mechanisms leading to MDR include enhancing drug efflux activity, advanced DNA damage repair functions, altered drug metabolism or reduced apoptosis (Gottesman et al., 2002; Eckford and Sharom, 2009). Among these mechanisms, the overexpression of adenosine triphosphate (ATP)-binding cassette (ABC) transporters resulted in increasing drug efflux is considered to be the predominant factor (Kathawala et al., 2015a; Kartal-Yandim et al., 2016).

The ABC transporters located on the cell membrane are efflux proteins and play important physiological and pharmacological roles (Dassa and Bouige, 2001). Currently, 49 distinct ABC transporters in which 48 of them are functional are split into seven subfamilies from ABCA to ABCG in humans (Stavrovskaya and Stromskaya, 2008; Eckford and Sharom, 2009). ABCB1 (P-glycoprotein, P-gp; multidrug resistance 1, MDR1), ABCG2 (breast cancer resistance protein, BCRP; mitoxantrone resistance, MXR), and ABCC1 (multidrug resistance protein 1, MRP1) are the most important ABC transporters and the overexpression of them render emergence of MDR in cancer cells (Dean, 2009; Li et al., 2016). ABCB1, as one of the major MDR contributors, is the most well-known and best characterized in the ABC transporter family. It was firstly discovered in 1976 and has been studied extensively (Juliano and Ling, 1976). It is generally distributed in bone marrow, blood-brain barrier (BBB), placenta, gut mucosa, liver, and kidney where it protects organ by preventing toxins from entering vital organs or facilitating the process of eliminating toxins via efflux (Sparreboom et al., 1997; Schinkel, 1999). Nevertheless, the overexpression of ABCB1 also results in reducing the intracellular accumulation of a wide variety of chemotherapeutic substrates including taxanes, *vinca* alkaloids, and anthracyclines (Tiwari et al., 2011; Schinkel and Jonker, 2012; Sodani et al., 2012). Thus, it is extremely urgent to investigate or develop novel compounds as inhibitors of ABCB1 to overcome the related MDR.

Bruton’s tyrosine kinase (BTK), a 77 kDa non-receptor tyrosine kinase, is a group of distinct cell surface receptors, especially B-cell antigen receptors (Mohamed et al., 2009). Since BTK regulates the activation, proliferation, maturation, differentiation and survival of B-cells, it has emerged as a novel molecular target in the treatment of leukemia and lymphoma of some B-cells (Mohamed et al., 2009; Singh et al., 2018). Furthermore, the study in prostate cancer cohort indicated that BTK knockdown selectively inhibited the growth of prostate cancer cells (Guo et al., 2014). In BTK highly expressed primary neuroblastoma samples, BTK inhibitor acalabrutinib elicited remarkable effect on inhibition of neuroblastoma tumorigenesis (Pikatan et al., 2020). New BTK inhibitors developed by indole derivatives also appeared for related cancer treatments (Rathi et al., 2017). Notably, BTK inhibitors had multiple off-target effects, especially ibrutinib (Gavriatopoulou et al., 2020; Metzler et al., 2020). Currently, ibrutinib, which was approved by Food and Drug Administration (FDA) for treating mantle cell lymphoma (MCL) and chronic lymphocytic leukemia (CLL) (Zhang et al., 2017), has been reported to have significant effect to improve the efficacy of chemotherapeutic drugs transported by ABCB1 transporter in ABCB1-overexpressing cells (Zhang et al., 2017).

Another BTK inhibitor, RN486, is a selective, and reversible inhibitor of BTK. It binds to the enzyme potently and competitively. In addition, RN486 is a preclinical drug and has the potential therapeutic benefits in treating rheumatoid arthritis (RA) in rodents and RA synovial tissue explants (Xu et al., 2012; Hartkamp et al., 2015). Moreover, in mouse models, RN486 attenuates systemic lupus erythematosus (SLE) by inhibiting B-cell activation, reducing the secretion of IgG anti-double-stranded DNA (anti-dsDNA), and impacting the effector function of autoantibodies (Mina-Osorio et al., 2013).

In this study, we evaluate whether the BTK inhibitor, RN486, has similar effect as ibrutinib to enhance the tumor suppression activity of chemotherapeutic agents and overcome ABCB1-mediated MDR.

## Materials and Methods

### Chemicals

RN486 was a gift from Chemie Tek (Indianapolis, IN, United States). Dulbecco’s modified Eagle’s Medium (DMEM), fetal bovine serum (FBS), penicillin/streptomycin, and 0.25% trypsin were obtained from Corning Incorporated (Corning, NY, United States). The monoclonal antibody of GAPDH (catalog number MA5-15738, lot number SA247966, clone GA1R), Alexa Fluor 488 conjugated goat anti-mouse IgG secondary antibody, bicinchoninic acid (BCA) assay Reagents, and enhanced chemiluminescence (ECL) Western blotting substrate were products from Thermo Fisher Scientific Inc. (Rockford, IL). The compounds cisplatin, colchicine, paclitaxel, doxorubicin, verapamil, the monoclonal antibodies for ABCB1 (catalog number P7965, lot number 067M4761V, clone F4), dimethyl sulfoxide (DMSO), 3-(4,5-dimethylthiazolyl)-2,5-diphenyltetrazolium bromide (MTT), Triton X-100, 4′,6-diamidino-2-phenylindole (DAPI), and paraformaldehyde, were purchased from Sigma-Aldrich (St. Louis, MO, United States). Horseradish peroxide (HRP)-conjugated rabbit anti-mouse IgG secondary antibody (catalog number 7076S, Lot number 32) were obtained from Cell Signaling Technology Inc. (Danvers, MA, United States). Bovine Serum Albumin (BSA) and 10 X phosphate buffer solution (PBS) were gained from VWR chemicals, LLC (Solon, OH, United States). [^3^H]-paclitaxel (15 Ci/mmol) was bought from Moravek Biochemicals, Inc. (Brea, CA, United States). All the other chemicals were purchased from Sigma Chemical Co. (St. Louis, MO, United States).

### Cell Lines and Cell Culture

The colchicine-induced ABCB1-overexpressing resistant cell line KB-C2 and its parental cell line, human epidermoid carcinoma KB-3-1, were used in our study (Wang et al., 2014). HEK293/pcDNA3.1, HEK293/ABCB1 were transfected human embryonic kidney HEK293 cells carried empty pcDNA3.1 vector and the vector containing ABCB1 gene, respectively. G418 (2 mg/ml) was used to select transfected cells (Kathawala et al., 2015b). DMEM medium containing 10% fetal bovine serum and 1% penicillin/streptomycin was prepared to culture all the mentioned cells with the condition of 37°C incubator having 5% CO_2_. All cells were used in experiments when they formed adherent monolayer with 60–80% cell confluence.

### Cytotoxicity and Reversal Experiments

The MTT assay was used in cell viability and reversal experiments as previously described (Lyall et al., 1987). The concentration of 5 × 10^3^ cells per well were seeded with 160 μl medium in 96-well plates and the plate was maintained in incubator overnight. In cytotoxicity experiment, the diluted concentrations of reversal reagent RN486 were administrated into different wells. In reversal experiment, various concentrations of RN486 were added 2 h prior to the addition of diluted concentrations of traditional chemotherapeutic drugs to specific wells. After incubation of 68 h, 20 μl MTT solution (4 mg/ml) was added to all the wells following further incubation of cells for 4 h. The supernatant of each well was discarded and 100 μl of DMSO was added to dissolve the formazan crystals. By using a UV/Vis Microplate Spectrophotometer (Fisher Sci., Fair Lawn, NJ, United States), the light absorbance was detected with 570 nm wavelength. IC_50_ value which is the concentration for 50% inhibition of cell viability was calculated as previously described (Ji et al., 2019a). Verapamil was used as a positive control inhibitor of ABCB1 transporter. Non-substrate drug of ABCB1, cisplatin, was used as a negative control chemotherapeutic drug in reversal experiment.

### Immunoblot Analysis

Based on the MTT assay, the concentration 3 μM of RN486 was used to treat the resistant cells with different time periods (0, 24, 48, and 72 h). The parental cells without RN486 were also incubated for 72 h. Then all the cells were lysed after twice washing of ice-cold PBS. The protein concentration was determined by BCA Protein Assay Kit (Thermo Scientific, Rockford, IL, United States). Same amounts of protein were loaded in sodium dodecyl sulfate polyacrylamide gel following the transfer of protein from the gel onto polyvinylidene fluoride (PVDF) membranes. To eliminate non-specific protein binding, membrane blocking was performed in TBST (Tris–buffered saline, 0.1% Tween 20) buffer with 5% non-fat milk for 2 h at room temperature. Afterward, the primary antibodies of ABCB1 (1:1000) and GAPDH (1:1000) were used to immunoblot against the membrane overnight at 4°C. The HRP-conjugated secondary antibody (1:1000) was applied against primary antibodies for 2 h at room temperature after the membrane was washed with TBST buffer. Next, three times washing of membrane was conducted in TBST buffer with the interval of 5 min. Chemiluminescent signal was obtained by the reaction between luminescent substrate ECL and secondary antibody. Protein quantification was analyzed by using ImageJ software.

### Immunofluorescence Assay

The immunofluorescence assay was carried out following the previous description (Cai et al., 2020). KB-3-1 and KB-C2 cells (1 × 10^4^) were seeded in 24-well plates followed by overnight culture at 37°C. Then, the cells were incubated with or without 3 μM RN486 at the different time periods (0, 24, 48, and 72 h) in designated wells. After being washed twice with ice-cold PBS, the fixation of cells was performed in wells by 4% formaldehyde for 15 min. The 0.25% Triton X-100 was used to treat cells for 15 min followed by the incubation with BSA (6% with PBS) for 1 h of blocking. Cells were incubated with primary monoclonal antibodies of ABCB1 (1:1000) overnight at 4°C. Later, Alexa Fluor 488 conjugated IgG secondary antibody (1:1000) was added in wells for 2 h in dark after the cells getting washed with PBS. The nuclei of cells were counterstained by DAPI solution. Fluorescence images were captured by Nikon TE-2000S microscope (Nikon Instruments Inc., Melville, NY, United States).

### [^3^H]-Paclitaxel Accumulation and Efflux Assay

As previously described (Fan et al., 2018; Cui et al., 2019b), accumulation and efflux assays were conducted to explore the antagonistic mechanism of RN486 by using a substrate of ABCB1, [^3^H]-paclitaxel. For [^3^H]-paclitaxel accumulation assay, KB-3-1 and KB-C2 cells with concentration of 1 × 10^5^ per well were inoculated into 24-well plates. On the next day, various concentrations of RN486 (0, 1, and 3 μM) and verapamil (3 μM) were added separately in both parental KB-3-1 and its resistant KB-C2 cell lines 2 h before adding [^3^H]-paclitaxel. After culturing the cells with [^3^H]-paclitaxel for 2 h, trypsin-EDTA was used to digest the cells and the cells were collected into 5 ml scintillation solution. For [^3^H]-paclitaxel efflux assay, distinct concentrations of RN486 (0, 1, and 3 μM) and verapamil (3 μM) were added 2 h before [^3^H]-paclitaxel. After culturing [^3^H]-paclitaxel, the supernatant was discarded and medium was added with the absence or presence of inhibitor. Finally, cells were collected at 0, 30, 60, and 120 min. By using Packard TRI-CARB1 190′A liquid scintillation analyzer (Packard Instrument, Downers Grove, IL, United States), the radioactivity of the samples at different time points were collected (Wang J. et al., 2020).

### ATPase Assay

The ATPase assay of ABCB1 transporter was carried out using PREDEASY ATPase assay kit (TEBU-BIO nv, Boechout, Belgium) as previously described (Cui et al., 2019b). Membrane (10 μg) was incubated with or without sodium orthovanadate (0.3 mM) in assay buffer. Then, RN486 (0–40 μM) was added. The reaction was initiated once adding 5 mM of ATP and terminated by adding 5% SDS solution. The amount of inorganic phosphate (Pi) was determined with a colorimetric method as described before (Ji et al., 2019b). Light absorbance was measured at 880 nm using a spectrophotometer.

### Molecular Docking of RN486 With Human ABCB1 Models

The RN486 3-D structure was established for docking simulation with a human ABCB1 model as previously described (Wang J. Q. et al., 2020). Human ABCB1 protein model 6QEX (ligand: paclitaxel, an ABCB1 ATPase stimulator) and 6QEE (ligand: zosuquidar, an ABCB1 ATPase inhibitor) were acquired from RCSB Protein Data Bank. Both models are inward-facing human ABCB1 with a resolution of 3.6 Å (6QEX) or 3.9 Å (6QEE) (Alam et al., 2019). AutoDock Vina (version 1.1.2) was used for docking calculations (Vina, 2010). Hydrogen atoms and partial charges were added using AutoDock Tools (ADT, version 1.5.4). Docking grid center coordinates were determined from the bound ligands provided in PDB files. The default settings were used for receptor/ligand preparation and docking. Based on the sorted affinity score (kcal/mol), the top-scoring pose was further analyzed and visualized.

### Statistics

The data are presented as the mean ± SD. One-way analysis of variance (ANOVA) followed by Dunnett’s *post hoc* test was used to analyze the significance. When *P* < 0.05, it is considered statistically significant difference between treatment groups with corresponding control group. All experiments were repeated at least three times.

## Results

### RN486 Enhanced the Efficacy of Anti-cancer Drugs in ABCB1-Overexpressing Cancer Cells

**FIGURE 1:**
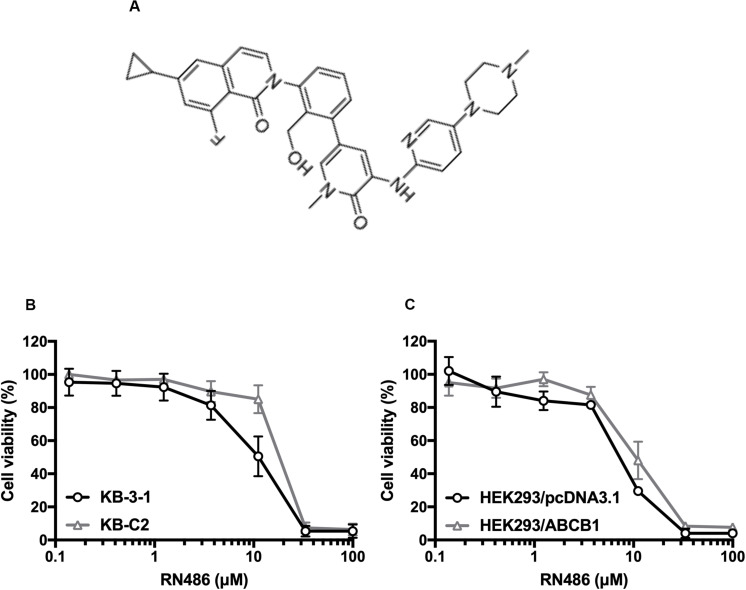
Chemical structure of RN486 and cell viability curves in parental and drug-selected or ABCB1-transfected cancer cells administered with RN486. **(A)** Chemical structure of RN486. **(B,C)** Concentration-viability curves for KB-3-1, KB-C2, HEK293/pcDNA3.1, and HEK293/ABCB1 cells administered with RN486. Cytotoxicity of RN486 was determined by MTT assay. Dots with error bars in the image represent mean ± SD with three independent assays.

The cytotoxicity of RN486 was firstly tested in various cell lines. The non-toxic concentrations (0.3, 1, and 3 μM) that less than IC_20_ (concentration for 20% inhibition) after 72 h-incubation were selected according to the results (Figure 1). The reversal data were shown in Table 1. Compared to parental KB-3–1 cells, the IC_50_ values of doxorubicin and paclitaxel were much higher in the resistant KB-C2 cells, which rendered high resistance-fold, by 101.4- and 707.3-fold, respectively. The IC_50_ values of doxorubicin and paclitaxel in KB-C2 cells were dramatically decreased with the addition of RN486 in a concentration-dependent manner. RN486 had greater reversal effect than verapamil at the same concentration (3 μM). The reversal experiments on the transfected HEK293/pcDNA3.1 and HEK293/ABCB1 cells were conducted to confirm that RN486 improves the efficacy of anticancer drugs by the interaction with ABCB1 transporter. As shown in Table 2, RN486 re-sensitized the ABCB1-overexpressing cell line HEK293/ABCB1 compared with the vector control cell line HEK293/pcDNA3.1. In this study, the well-known ABCB1 inhibitor verapamil was served as positive control of the reversal reagent and cisplatin was used as negative control of anticancer drug since it was not a substrate of ABCB1.

**TABLE 1:** The reversal effects of RN486 on ABCB1-mediated MDR in drug-selected resistant cells.

Treatment	IC_50_ ± SD^a^ (RF^b^)	
	**KB-3-1 (μM)**	**KB-C2 (μM)**
**Doxorubicin**	0.019 ± 0.004 (1.00)	1.898 ± 0.256 (101.39)
+RN486 (0.3 μM)	0.020 ± 0.008 (1.09)	0.434 ± 0.159 (23.19)*
+RN486 (1 μM)	0.019 ± 0.004 (1.00)	0.029 ± 0.009 (1.54)*
+RN486 (3 μM)	0.013 ± 0.003 (0.78)	0.012 ± 0.002 (0.63)*
+Verapamil (3 μM)	0.013 ± 0.002 (0.71)	0.036 ± 0.008 (1.93)*
**Paclitaxel**	0.003 ± 0.001 (1.00)	1.820 ± 0.198 (707.30)
+RN486 (0.3 μM)	0.003 ± 0.001 (1.12)	1.427 ± 0.321 (554.31)
+RN486 (1 μM)	0.003 ± 0.001 (1.11)	0.059 ± 0.015 (22.76)*
+RN486 (3 μM)	0.003 ± 0.001 (1.23)	0.003 ± 0.001 (1.30)*
+Verapamil (3 μM)	0.002 ± 0.001 (0.82)	0.062 ± 0.019 (23.96)*
**Cisplatin**	2.572 ± 0.684 (1.00)	4.108 ± 2.134 (1.60)
+RN486 (0.3 μM)	2.731 ± 0.637 (1.06)	4.460 ± 1.188 (1.73)
+RN486 (1 μM)	3.094 ± 1.140 (1.20)	3.718 ± 1.687 (1.45)
+RN486 (3 μM)	2.871 ± 0.817 (1.12)	4.476 ± 1.799 (1.74)
+Verapamil (3 μM)	2.865 ± 1.278 (1.11)	3.777 ± 0.765 (1.47)

*^*a*^IC_50_ values represent mean ± standard deviation (SD) acquired from at least three independent experiments. ^*b*^Resistance fold (RF) was calculated from dividing the IC_50_ values of parental or resistant cells in the presence or absence of RN486 or positive control inhibitor verapamil by the IC_50_ values of parental cells without reversal reagent. ^∗^*p* < 0.05 versus control treatment in the absence of reversal reagent.*

**TABLE 2:** The reversal effects of RN486 on ABCB1-mediated MDR in ABCB1-transfected cells.

Treatment	IC_50_ ± SD^a^ (RF^b^)	
	HEK293/pcDNA3.1 (μM)	HEK293/ABCB1 (μM)
**Doxorubicin**	0.061 ± 0.010 (1.00)	3.163 ± 0.472 (51.88)
+RN486 (0.3 μM)	0.057 ± 0.007 (0.93)	2.631 ± 0.210 (43.15)
+RN486 (1 μM)	0.052 ± 0.014 (0.85)	0.176 ± 0.084 (2.88)*
+RN486 (3 μM)	0.048 ± 0.003 (0.79)	0.066 ± 0.018 (1.08)*
+Verapamil (3 μM)	0.069 ± 0.022 (1.14)	0.259 ± 0.068 (4.25)*
**Paclitaxel**	0.048 ± 0.009 (1.00)	2.970 ± 0.780 (61.29)
+RN486 (0.3 μM)	0.049 ± 0.011 (1.01)	1.639 ± 0.447 (33.82)
+RN486 (1 μM)	0.041 ± 0.007 (0.85)	0.321 ± 0.088 (6.62)*
+RN486 (3 μM)	0.037 ± 0.005 (0.77)	0.040 ± 0.021 (1.13)*
+Verapamil (3 μM)	0.040 ± 0.004 (0.82)	0.303 ± 0.057 (6.25)*
**Cisplatin**	4.134 ± 1.507 (1.00)	5.341 ± 0.791 (1.29)
+RN486 (0.3 μM)	4.259 ± 1.128 (1.03)	6.031 ± 0.698 (1.46)
+RN486 (1 μM)	3.894 ± 0.753 (0.94)	6.459 ± 0.952 (1.56)
+RN486 (3 μM)	4.480 ± 0.767 (1.08)	5.667 ± 0.298 (1.37)
+Verapamil (3 μM)	4.415 ± 1.955 (1.07)	4.693 ± 0.497 (1.14)

*^*a*^IC_50_ values represent mean ± standard deviation (SD) acquired from at least three independent experiments. ^*b*^Resistance fold (RF) was calculated from dividing the IC_50_ values of parental or resistant cells in the presence or absence of RN486 or positive control inhibitor verapamil by the IC_50_ values of parental cells without reversal reagent. ^∗^*p* < 0.05 versus control treatment in the absence of reversal reagent.*

### RN486 Did Not Significantly Affect the Expression Level of ABCB1 and Subcellular Localization in ABCB1-Overexpressing Cancer Cells

**FIGURE 2:**
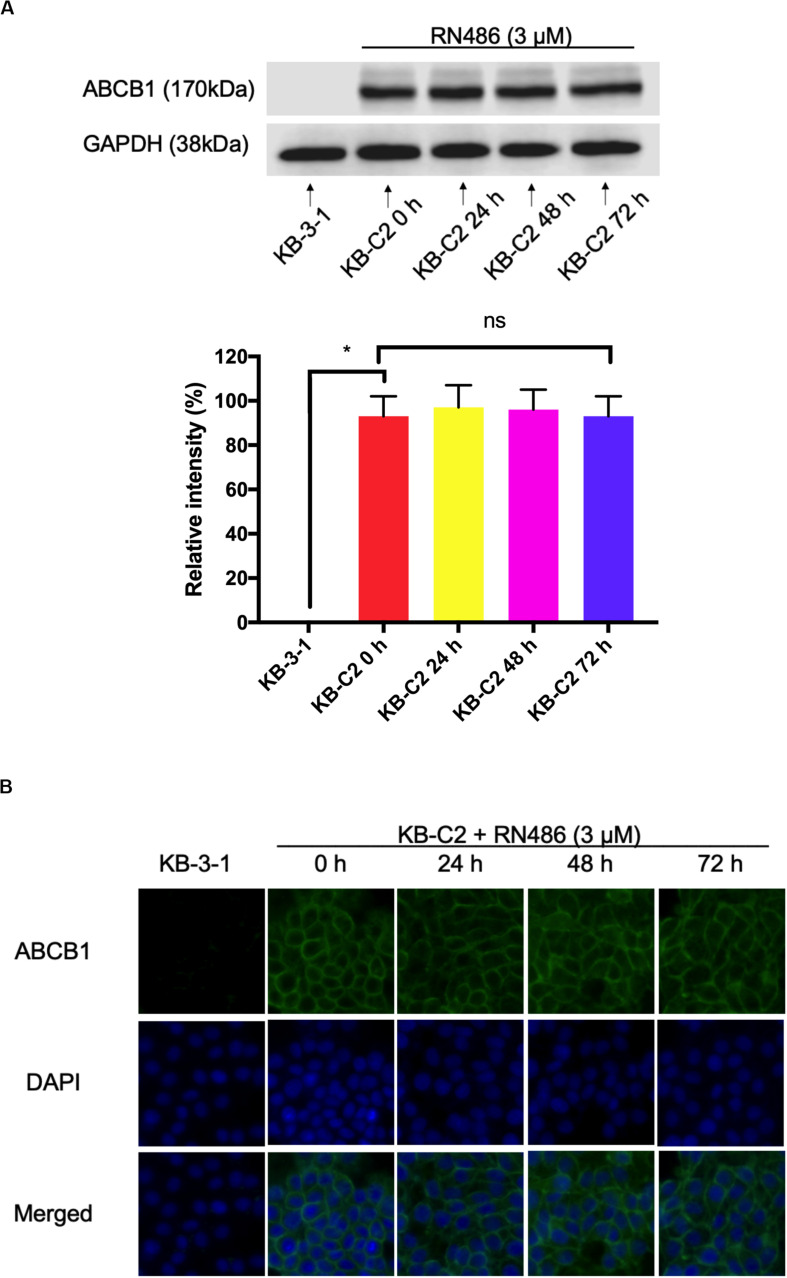
RN486 did not change the protein expression and location of ABCB1. **(A)** ABCB1 expression level detected in KB-C2 cells with the treatment of 3 μM RN486 for 0, 24, 48, and 72 h. **(B)** Immunofluorescence on the subcellular localization of ABCB1 transporter with the treatment of 3 μM RN486 for 0, 24, 48, and 72 h. Values are mean ± SD, representative of three independent assays. KB-3-1 acts as control group. NS represents no significance.

From the above MTT assay, RN486 indeed antagonized ABCB1-mediated MDR. The possible reversal mechanism may include down-regulation of the protein or change the localization of protein. We carried out Western blotting and immunofluorescence assay to look into these possibilities. The optimal concentration of RN486 at 3 μM was selected in both assays and KB-3-1 cells were used as negative control cell line. It was found that the expression level of ABCB1 in KB-C2 cells did not change significantly with these treatments after being incubated for different time (0, 24, 48, and 72 h) (Figure 2A). Moreover, our results showed that the expression of ABCB1 protein remained on the cell membrane of KB-C2 cells without trans-localizing to the cytoplasm or other organelles during 24–72 h treatment with RN486 (Figure 2B). These results indicated that RN486 reversed ABCB1-mediated MDR are not related to the down-regulation of ABCB1 protein or alteration of its subcellular localization.

### RN486 Increased the Intracellular Accumulation of [^3^H]-Paclitaxel in ABCB1-Mediated MDR Cancer Cells

**FIGURE 3:**
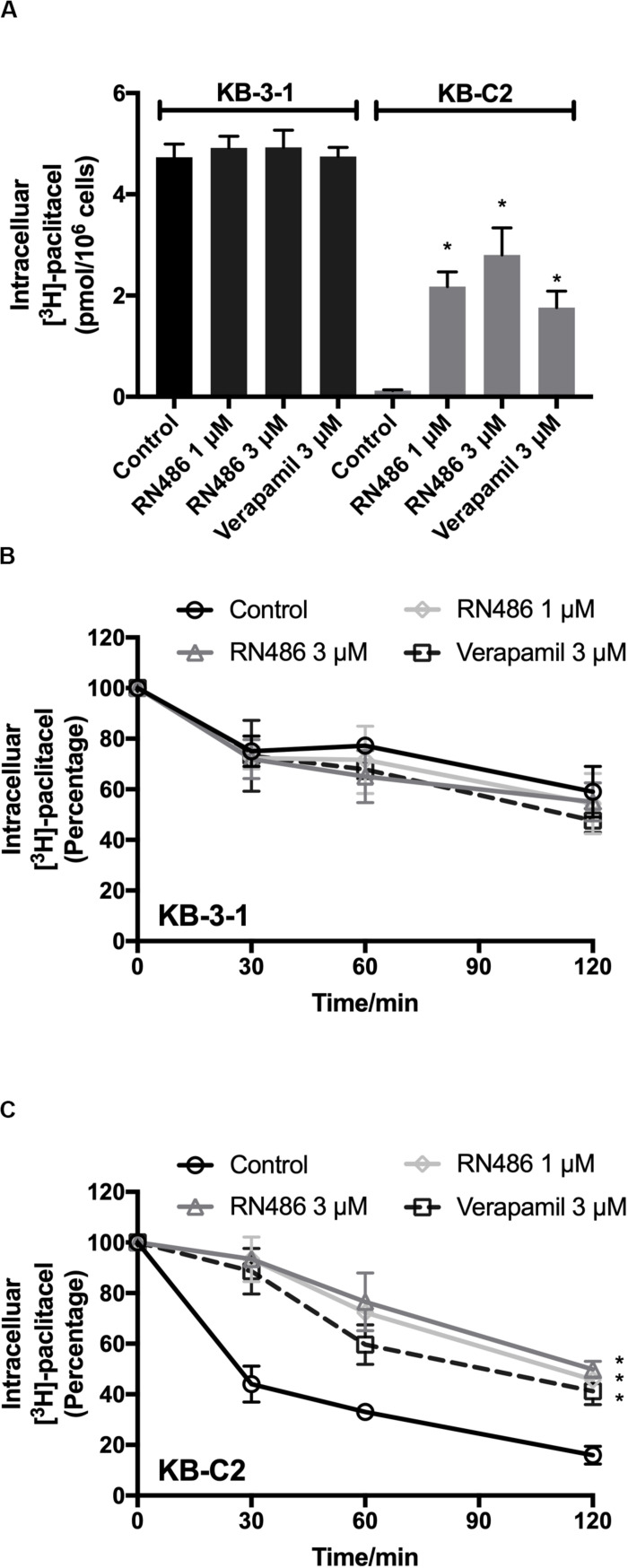
Administered RN486 resulting in the increase of intracellular [^3^H]-drug accumulation and the blockade of efflux activity of ABCB1 transporter in ABCB1-overexpressing cancer cells. **(A)** The effect of RN486 on the accumulation function of [^3^H]-paclitaxel in ABCB1-mediated MDR cell lines. **(B,C)** The effects of RN486 on the efflux activity of [^3^H]-paclitaxel in parental KB-3-1 and its drug-selected KB-C2 cells. Verapamil (3 μM) was a positive control. Results shown as mean ± SD, three independent experiments were performed. *p < 0.05 versus control treatment.

To further evaluate the function of RN486 when it was used together with an anticancer drug, we performed the [^3^H]-paclitaxel accumulation assay in KB-3-1 and KB-C2 cells. The concentrations of [^3^H]-paclitaxel were accumulative from the induction of it to the time of cell detachment. As shown in Figure 3A, RN486 significantly increased the intracellular accumulation of [^3^H]-paclitaxel in the drug resistant KB-C2 cells, especially when the concentration was 3 μM. In addition, the increased levels of [^3^H]-paclitaxel accumulation in cells were more significant in those treated with both 1 and 3 μM RN486 (2.17 and 2.80 pmol per 10^6^ cells) compared to those treated with 3 μM verapamil (1.76 pmol per 10^6^ cells) in resistant KB-C2 cells. RN486 did not significantly interfere the accumulation of [^3^H]-paclitaxel in parental KB-3-1 cells (0.12 pmol per 10^6^ cells). These results suggested that RN486 may inhibit the function of ABCB1 transporter which leads to increased drug accumulation inside the cells.

### RN486 Blocked the Efflux Activity Mediated by ABCB1 Transporter in ABCB1-Overexpressing Cancer Cells

The efflux assay was performed after accumulation assay to investigate the antagonization mechanism of RN486 in cancer cells. The concentrations of [^3^H]-paclitaxel in the cells were transient at different time points. It was found that RN486 did not apparently affect efflux of [^3^H]-paclitaxel in KB-3-1 cells (Figure 3B). However, the efflux activity was significantly decreased by RN486 in KB-C2 cells (Figure 3C). At the time point of 120 min, comparing the concentration at 0 min, the percentage of intracellular [^3^H]-paclitaxel decreased to 16% in control group. For the treatment groups with 1, 3 μM RN486, and 3 μM verapamil, the percentage reduced to 45.76, 49.87, and 41.21%, respectively. The results of high percentage of retained [^3^H]-paclitaxel indicated that RN486 can interact with ABCB1 transporter and result in blocking the efflux activity of ABCB1-overexpressing cell line.

### The Effect of RN486 on ATPase Activity of ABCB1

**FIGURE 4:**
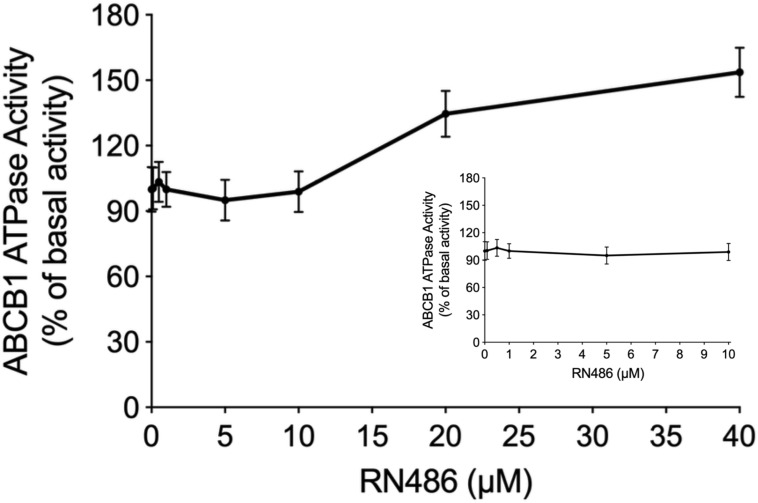
The effect of RN486 on the ATPase activity of ABCB1. Different concentrations (0–40 μM) of RN486 affected the ATP hydrolysis process, which mediated by ATPase of ABCB1. The inset graph demonstrates the effect of 0–10 μM RN486 on the ATPase activity of ABCB1. Data are mean ± SD of three independent assays.

The energy used to efflux the substrate drugs of ABCB1 from the intracellular side to the extracellular side against the concentration gradient was provided by hydrolysis of ATP, which means the ATPase catalytic activity of ABCB1 transporter was of great significance. To further evaluate the effect of RN486 on the basal activity of ABCB1 ATPase, different concentrations of RN486 (0–40 μM) were incubated with the ABCB1-riched membranes using the ATPase assay kit. As shown in the results (Figure 4), no significant stimulatory or inhibitory impact was found by introducing lower concentrations of RN486, however, the stimulation effect of RN486 to ATPase activity of ABCB1 became boosted with higher concentrations of RN486, and the maximal stimulation reached up to 154% of basal activity. The stimulatory effect of RN486 on ABCB1 reached half-maximum (EC_50_) at the concentration of 13 μM.

### Docking Simulation of RN486 in the Drug-Binding Pocket of Human ABCB1

**FIGURE 5:**
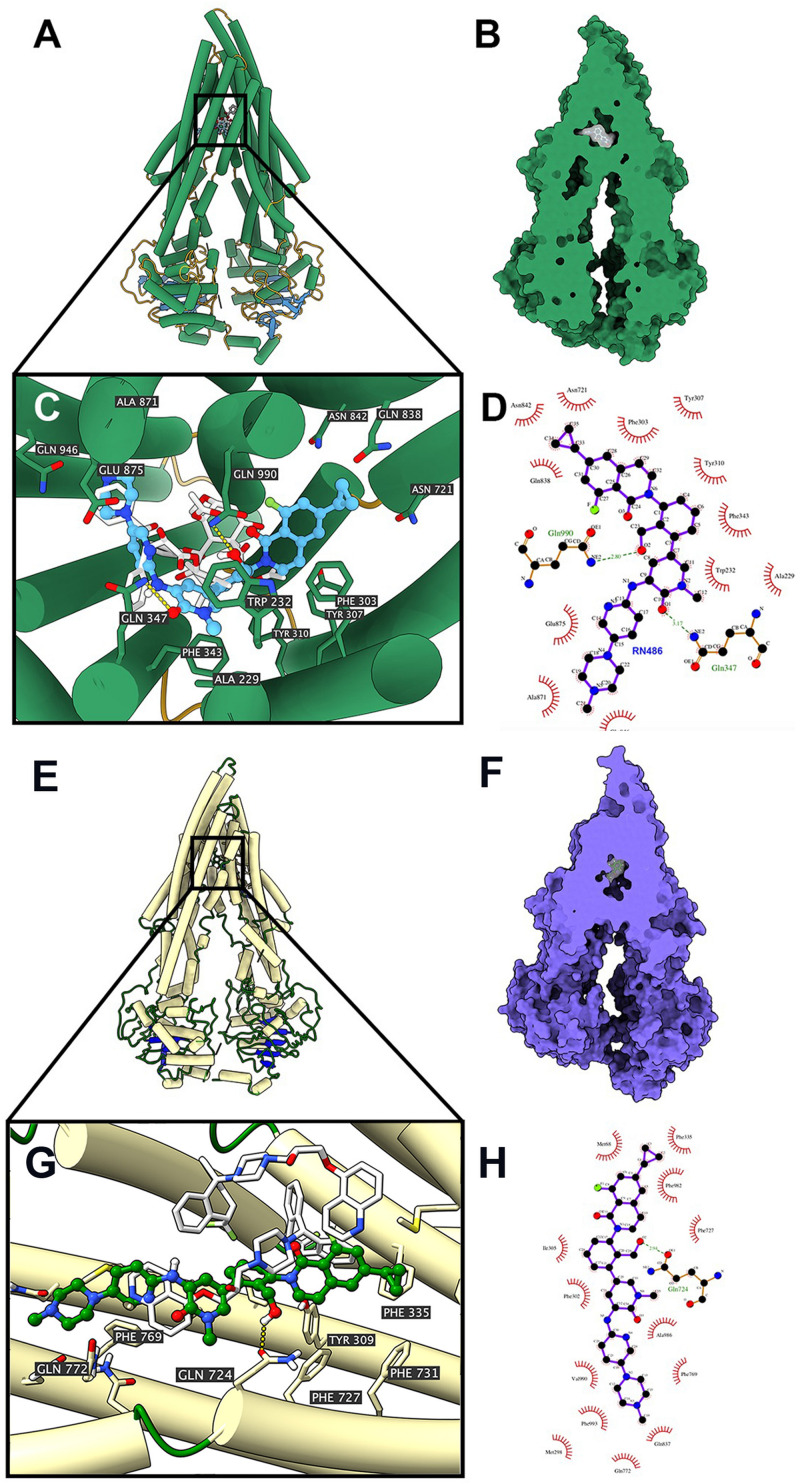
Interaction between RN486 and human ABCB1 protein. **(A)** Overview of paclitaxel and the best-scoring pose of RN486 in the drug-binding pocket of ABCB1 protein (6QEX). ABCB1 was displayed as colored tubes and ribbons. RN486 and paclitaxel were displayed as colored sticks. Carbon: blue (RN486) or white (paclitaxel); oxygen: red; nitrogen: blue; fluoride: green. **(B)** Interactions between RN486 and ABCB1 binding pocket with the protein surface of ABCB1 displayed. **(C)** Details of interactions between RN486 and ABCB1 binding pocket. Hydrogen bonds were displayed as yellow dash lines. **(D)** 2D RN486-ABCB1 interaction. Important amino acids were displayed as red arcs, and the green dash line with number indicates hydrogen bond with bond length. Carbon: black; oxygen: red; nitrogen: blue; fluoride: green. **(E)** Overview of zosuquidar and the best-scoring pose of RN486 in the drug-binding pocket of ABCB1 protein (6QEE). ABCB1 was displayed as colored tubes and ribbons. RN486 and zosuquidar were displayed as colored sticks. Carbon: green (RN486) or white (zosuquidar); oxygen: red; nitrogen: blue; fluoride: light green. **(F)** Interactions between RN486 and ABCB1 binding pocket with protein surface of ABCB1 displayed. **(G)** Details of interactions between RN486 and ABCB1 binding pocket. Hydrogen bonds were displayed as yellow dash lines. **(H)** 2D RN486-ABCB1 interaction. Color codes are same as **(D)**.

According to the ATPase assay result, RN486 showed stimulatory effect at higher concentrations. Consequently, we applied docking simulation in ATPase-stimulator (substrate) binding site of human ABCB1 protein (6QEX). The results showed that RN486 docked into the substrate site with an affinity score of -9.5 kcal/mol. Details of ligand-receptor interaction were displayed in Figures 5A–D. For the substrate-binding site, hydrophobic interactions played a crucial role in facilitating the binding of RN486 to the ABCB1 protein. RN486 was positioned and stabilized in the hydrophobic cavity formed by Ala229, Trp232, Phe303, Tyr307, Tyr310, Phe343, Asn721, Gln838, Asn842, Ala871, Glu875, and Gln946. Besides, the formation of hydrogen bond with Gln990 contributed to the stability of the hydroxide group of RN486 and the pyridine group of RN486 got stabilized by a formed hydrogen bond at Gln347. Additionally, RN486 didn’t show significant stimulatory or inhibitory effects on ABCB1 ATPase at lower concentrations, which could be due to the counter-effect of stimulator and inhibitor. Therefore, we also performed docking simulation of RN486 and the ATPase inhibitor binding site of human ABCB1 (6QEE). The results showed that RN486 docked into the inhibitor binding site with an affinity score of −9.1 kcal/mol, which is similar to the score of substrate-binding complex. Details of ligand-receptor interaction were displayed in Figures 5E–H. For inhibitor binding site, hydrophobic interactions also played a key role in promoting the binding of RN486 to the ABCB1 protein. RN486 was positioned and stabilized in the hydrophobic cavity formed by Met68, Phe335, Phe982, Phe727, Ala986, Phe769, Phe993, Val990, Phe302, and Ile305. Moreover, the hydrogen bond formed with Gln724 made the hydroxide group of RN486 steady.

## Discussion

Overexpression of ABC transporter leading to MDR still remains a major barrier of successful chemotherapy (Chen et al., 2016; Amawi et al., 2019). ABCB1, as one of the most important ABC transporters, is widely distributed in the body (Linton, 2007). The protective function of ABCB1 transporter is to pump out xenobiotics. However, it could induce MDR if ABCB1 transporter gets overexpressed in tumor cells. The potential approach to improve the efficacy of chemotherapeutic drugs and overcome ABCB1-mediated MDR is the combination of reversal agents with anticancer drugs. Several studies showed the feasibility of small-molecule combination therapy. The reversal agents including breakpoint cluster region-abelson (BCR-ABL) inhibitors imatinib (STI571) and ponatinib (Lei et al., 2006; Sen et al., 2012), epidermal growth factor receptor (EGFR) inhibitors lapatinib (GW-572016) and gefitinib (Kitazaki et al., 2005; Dai et al., 2008), vascular endothelial growth factor receptor (VEGFR) inhibitors vandetanib and motesanib (Zheng et al., 2009; Wang et al., 2014). However, although it was reported that ibrutinib could antagonize MDR mediated by ABCB1 (Zhang et al., 2017), the study on other BTK inhibitors in reversing MDR mediated by ABCB1-overexpression is still lacking.

RN486 is a selective, and reversible inhibitor of BTK. As a preclinical drug, it has been reported to have satisfactory anti-inflammatory and bone-protective effects in rodents (Xu et al., 2012). Several studies have shown that RN486 offers a novel attractive therapeutic alternative to the current RA therapy (Zhao et al., 2015a, b; Zhao et al., 2015c). However, there is no reports related to the antitumor effect of RN486 at present. Here we reported RN486 could surmount ABCB1-mediated MDR effectively in drug resistant cancer cells.

In this study, MTT assays were firstly designed to evaluate the possibility of cytotoxicity induced by RN486. According to the results, non-toxic concentrations (0.3, 1, and 3 μM) of RN486 were selected in reversal experiments. Our data showed RN486, without interfering the parental KB-3-1 and HEK293/pcDNA3.1 cells, dramatically re-sensitized their resistant KB-C2 and HEK293/ABCB1 cells to ABCB1 substrates paclitaxel and doxorubicin, which indicated the great reversal effect of RN486 in ABCB1-overexpressing cells. Furthermore, the reversal effect of 3 μM RN486 was superior to the same concentration of positive control inhibitor of ABCB1 verapamil, suggesting that RN486 was more potent and effective than verapamil. Additionally, in the future, it is also valuable to evaluate the reversal effect of RN486 to other ABC transporters such as ABCG2 or ABCC1.

In order to get a better understanding of the reversal mechanism of RN486, Western blotting and immunofluorescence assay were carried out since the regulated expression and changed subcellular localization of ABCB1 transporter may happen after RN486 treatment. Our results suggested that RN486 did not affect the expression level of ABCB1 transporter in all resistant KB-C2 cells treated with RN486 (3 μM) up to 72 h. Similar results were found in immunofluorescence assay. After incubation with RN486 (3 μM) up to 72 h, the ABCB1 transporter in resistant cells still located on the cell membrane without alteration. All these findings indicated that RN486 does not change expression level and subcellular localization of ABCB1 transporter. However, further experiments are warranted in the future to investigate the impact of longer treatment period or higher concentration of RN486 to ABCB1 transporter and also the effect of RN486 to other proteins that may be involved in the RN486 reversal mechanism.

Moreover, accumulation and efflux assays were conducted to have a better understanding of the antagonized function of RN486 to ABCB1-mediated MDR. Based on our results, in ABCB1-overexpressing KB-C2 cells, intracellular substrate concentration was dramatically increased by incubated with RN486 in the accumulation assay and the efflux function of ABCB1 was blocked by RN486. Nevertheless, RN486 has no effect on parental KB-3-1 cells. These results were consistent with our reversal studies, which RN486 has significant effect on reversing ABCB1-mediated MDR. The results also illustrate that by inhibiting transporter efflux activity, co-administered RN486 increases intracellular accumulation of substrate which enhances the efficacy of chemotherapy.

Energy supplemented by ATP hydrolysis plays an important role in the activity of ABCB1 transporter. The substrates or inhibitors of ABCB1 transporter may affect ATPase activity (Zhang et al., 2018; Ji et al., 2019a). The decreased trend of ATPase activity would be observed when the drug serves as an ATPase inhibitor since it inhibits the function of ABCB1 transporter, however, the substrate would stimulate the ATPase at substrate-binding site which provides energy to facilitate drug efflux. Thus, we performed ATPase assay to evaluate the effect of RN486 on ATPase function of ABCB1. From our results, the ATP hydrolysis of ABCB1 was less affected when the concentrations of RN486 was lower, but higher concentrations of RN486 exhibited the distinct stimulatory effect on ATPase of ABCB1 in a dose-dependent manner with a maximal level of 1.54-fold. These results suggested that RN486 neither stimulate nor inhibit the ATPase activity at lower concentrations. However, RN486 with higher concentrations may bind substrate-binding site of ABCB1, which granted its stimulatory effect on ATPase activity of ABCB1. It should be noted that some of ABC transporter substrate-drugs could competitively occupy the substrate-binding site resulting in inhibited efflux of certain substrates (Giri et al., 2009). RN486 may act on ABCB1 with the same mechanism. Furthermore, the cytotoxicity experiments excluded RN486 is a substrate of ABCB1. In addition, even though some ABCB1 inhibitors were identified as stimulators in ATPase assay of ABCB1, ABCB1 overexpression might not necessarily cause drug resistance to those inhibitors (Cui et al., 2019a; Wu et al., 2020). The reasons for unchanged ATPase activity with 0-10 μM RN486 may vary included additional interactions between RN486 with inhibitor binding site. As a wide-ranging used technique in structural molecular biology, the molecular docking study was carried out to further investigate the affinity of RN486 to the ABCB1 substrate and inhibitor binding sites. The results indicated that RN486 has high affinity to both transmembrane ATPase-stimulator (substrate) binding site and inhibitor binding site with the affinity score of −9.5 and −9.1 kcal/mol. Hydrophobic interactions between RN486 and ABCB1 protein contributed to the stability of RN486 to the hydrophobic cavity. Unlike erdafitinib (Feng et al., 2020) and MK-8776 (Cui et al., 2019b), RN486 preferred to bind both substrate-binding site and inhibitor binding site which indicated more complicated conformation changes emerged in the interaction of RN486 with human ABCB1 model. At the substrate-binding pocket of ABCB1 transporter, RN486 may supersede other anti-cancer drugs that are substrates of ABCB1 resulting in stimulation of ATPase activity. This binding may always exist and was dominant at higher concentrations of RN486. When the concentration of RN486 was lower, the binding of RN486 with inhibitor binding site probably also presented which restrained the activity of ATPase, the stimulatory and inhibitory effects of RN486 were neutralized, which in turn interpreted the flat curve of ATPase activity with 0–10 μM RN486 (Figure 4). In addition, both binding interactions between RN486 and human ABCB1 protein worked in blocking the efflux of anti-cancer drugs from ABCB1, which leading to the increased intracellular concentration of chemotherapeutic drugs like paclitaxel and rendering RN486 of potential reversal effect.

In conclusion, this study elucidated that the reversal mechanism of RN486 to ABCB1-mediated MDR is to inhibit anti-cancer drugs being pumped out by ABCB1 transporter without obstructing the expression and subcellular localization of ABCB1 protein. The ATPase assay and molecular docking study offered more details about possible interactions between RN486 and ABCB1 protein. Future studies may focus on other mechanisms of action of RN486 to ABCB1 transporter and *in vivo* tumor xenograft model. The combination therapy using RN486 with ABCB1 substrate chemotherapeutic drugs may offer an alternative approach to overcome ABCB1-mediated MDR.

## Data Availability Statement

All datasets presented in this study are included in the article/supplementary material.

## Author Contributions

Z-SC, LL, and WF designed the experiments. X-DD wrote the original draft manuscript. Z-NL and Q-XT reviewed the manuscript. X-DD, MZ, WF, J-QW, Z-NL, Q-XT, Y-DL, and XM performed the experiments. WF and LL analyzed the data. Z-SC and WF edited the manuscript. All authors discussed the data and approved the final manuscript.

## Conflict of Interest

The authors declare that the research was conducted in the absence of any commercial or financial relationships that could be construed as a potential conflict of interest.
